# Data-fed, needs-driven: Designing analytical workflows fit for disease surveillance

**DOI:** 10.3389/fvets.2023.1114800

**Published:** 2023-01-27

**Authors:** Fernanda C. Dórea, Flavie Vial, Crawford W. Revie

**Affiliations:** ^1^Department of Disease Control and Epidemiology, National Veterinary Institute, Uppsala, Sweden; ^2^Animal and Plant Health Agency, Sand Hutton, United Kingdom; ^3^Department of Computer and Information Sciences, University of Strathclyde, Glasgow, United Kingdom

**Keywords:** big data, epidemiology, decision support system, syndromic surveillance, data-driven surveillance

## Abstract

Syndromic surveillance has been an important driver for the incorporation of “big data analytics” into animal disease surveillance systems over the past decade. As the range of data sources to which automated data digitalization can be applied continues to grow, we discuss how to move beyond questions around the means to handle volume, variety and velocity, so as to ensure that the information generated is fit for disease surveillance purposes. We make the case that the value of data-driven surveillance depends on a “needs-driven” design approach to data digitalization and information delivery and highlight some of the current challenges and research frontiers in syndromic surveillance.

## 1. Introduction

The continuous and systematic collection and analysis of health-related data–a practice coined syndromic surveillance (SyS)–has gained momentum in public health since the turn of the century, buoyed by the putative benefit that SyS will allow detection of disease outbreaks or other public health trends earlier than traditional surveillance which relies on laboratory test results or clinical diagnoses.

“Big data analytics” is now recognized as a term referring not to the size of the data handled, but to the development in technologies needed to extract information from raw data, in an evolving and complex context (1). In animal health surveillance, this means specifically being able to convert data into actionable information for decision-makers tasked with disease prevention, detection and control. In 2011, Fricker (2) provided a broad overview of the issues related to the use of (digital) biosurveillance in practice. We highlight here his emphasis on the need to give more attention to system design, to ensure that the right information is available at the right time and in the right place to inform animal health actions.

By 2011, the idea of incorporating SyS methods into animal health surveillance systems were being more widely discussed (3). An intensive exploration of various sources of data ensued, as documented in reviews in 2013 (4), 2015 (5) and 2016 (6). The various initiatives documented in these reviews tended to share a focus on specific, individual streams of data. In these early stages, exploration focused on the methodological aspects of converting health events and other data streams into time-series that could be subjected to temporal aberration detection algorithms (TADA), and on validating the statistical analyses.

Ten years later, research into what Fricker had called the “operational challenge of biosurveillance” (ensuring statistical performance) has developed extensively across a range of veterinary SyS initiatives. But how close are we to achieving his view of a surveillance system that is designed to take into account stakeholders' needs and that produces the actionable information needed to support decision-making in practice?

## 2. Materials and methods

Power (7) identified three main characteristics of a decision support system (DSS): it should facilitate the decision-making process; it should *support* rather than *automate* decision making; and be able to respond quickly to the changing needs of decision-makers. The typical components of a DSS are the same as those we previously outlined for a data-driven surveillance framework (8)–data acquisition, analyses models and user interface.

There is no specific technical description for a DSS, which should be “defined in terms of the context and use” (9). System development can therefore only be successful if the users are explicitly involved. Sprague (10) argues, however, that not even the decision makers can anticipate the functional requirements of the system, as their needs are constantly changing, and the process of decision making itself can be altered by the system. He suggested that a DSS cannot be developed using the traditional “analysis, design, construction, implementation” cycle. Instead, these steps should be combined into a single step, which is iteratively repeated. The simplest system is built and delivered to users, and their feedback is continuously captured and incorporated into the DSS.

We reflect on some open research questions and the associated challenges these bring to SyS implementation, and suggest how some of those could be addressed using this simple DSS approach, which asks a single question: “how can this information improve the decision process of the final user?”.

In line with our view that early disease detection is too narrow a goal for data digitalization (8), we borrow the term syndromic surveillance for its established connotation as the “continuous monitoring of health data,” though our discussion considers surveillance of both exotic and endemic diseases.

We anchor our discussion around three main complementary examples:

(A) time-series of laboratory tests submissions, representing “typical” SyS;

(B) on-farm records relating to reproductive events in pigs, as an example of the still under-explored use of production data within SyS;

(C) food-borne surveillance as a One-Health example; specifically, the monitoring of gastrointestinal illness in humans and Campylobacter positive slaughter batches of chickens.

## 3. Results and discussion

### 3.1. Data acquisition

Most of the early SyS work was data-driven, i.e., focused on data that was relatively easy for system developers to access. Working example (A) is a typical case, where data owners, analysts and decision-makers all sit within the same organization (e.g., a national veterinary service). When data access is treated as a main impediment to further system development, only the needs of a subset of animal health stakeholders are considered. However, the majority of health-related data is collected by entities within the “animal health” network (e.g., industry groups) whose interests are different from those who are trying to draw actionable inferences from those data. For example, reproductive inefficiency will primarily be considered from an economic profitability standpoint by the manager of a dairy operation. That same increase in the number of abortions may be perceived by the veterinarian as an indicator of some underlying health issue in the herd. The regional veterinary services, which have received notifications in the previous 2 weeks of a large number of calves born with congenital deformities, may interpret this further as additional suspicion of a regional Bovine Viral Diarrhea outbreak which may require the enactment of an eradication scheme.

As a result, the field has started to move away from the pre-conception that data centralization is necessary to conduct population level surveillance. The technology of data federation allows the distribution of queries and models from a central location/body to the data nodes in a stakeholder network, rather than data having to flow in the opposite direction. In this “code to data” scenario (as opposed to the traditional “data to code”), data interoperability is prioritized over data harmonization. We have previously addressed this discussion and highlighted the importance of ontologies as a research priority (8).

Some surveillance systems may need to fulfill the decision support needs of the individual data providers themselves as well as those of the (non-data generating but policy-making) central node in the network. System design for implementation in the case of example (B) will require in the first instance the elicitation of the farmers'/associations' management requirements (i.e., their motivation to join the DSS). More research will subsequently be required around the technology available to deliver a system that analyses and delivers information at source, while sending only limited signals back to the network. Finding a balance between keeping farmers data as private as they wish, while collecting enough information to add value to decisions at a broader population level will require further discussions, with active farmer involvement.

The One Health example (C) represents yet another complex network of stakeholders. In this case, it is typical for separate central governmental bodies to have access to different data sets at the population level. The obligations of animal health and public health agencies to safeguard the identity of animal owners and individuals, respectively, may prevent data sharing between agencies at a high level. These data sources may be accessible, but can rarely be readily integrated. This is not an issue to solve with data management technology, but rather a feature to incorporate explicitly in DSS implementation, and we address this in the data analyses section below.

### 3.2. Syndromic indicators

SyS is mainly based on time-series analyses. The creation of a time-series is straightforward when data providers record the health events of interest in discrete time slots (commonly, days or weeks), as in examples (A) and (C): number of tests, number of cases, etc. per time unit.

Production data are recorded continuously on farms during normal activities, and events recorded are not necessarily associated with any health hazard. As such the events of “syndromic” interest must be defined, and metrics to determine their occurrence developed (11). Some production data may lose value if aggregated according to different time unit. In example (B), consider for instance the recording of the date of farrowing for each individual sow. The analyses may aggregate the number of farrowings per week in a particular farm, or report the average number of farrowings per sow per year. However, reproductive health may be better monitored by length of pregnancy, and farm management may thus be more interested in the time between two farrowings. A series where every farrowing is a new observation, and the value of the observation corresponds to the “number of days between farrowings,” is a continuous time-series. Observations are not grouped in any particular unit of time, as in the discrete time-series that SyS are typically designed to handle. Control charts, commonly used in SyS, were originally designed to monitor industrial processes that more closely resemble continuous time-series, so the application of TADA to these types of series is not a bottleneck. The challenges for their incorporation into automated monitoring systems are rather related to the definition and interpretation of outputs, and the large number of potential time-series that must be evaluated. We address system outputs in more detail below.

### 3.3. Data analyses

Aberration detection within single time-series has been intensively explored in SyS. When TADA are applied individually to time-series that represent counts of one type of syndrome, from one source, as is typical in (A), their use in practice will depend on resolving two main questions: how should we interpret alarms, that is, how to decide when an alarm deserves action?; and how can we best combine the evidence from multiple series? The answer to the first question almost certainly depends on the second, as single alarms are likely meaningless until placed within their broader context.

The need to combine evidence from multiple data streams has been addressed and reviewed before in both human (12) and animal health surveillance (13). However, the statistical solutions to monitoring multiple parallel time-series only solve a limited part of the problem. They are applicable in typical cases such as example (A), when a same source can produce multiple time-series aligned in time, or data for the same syndrome is coming from multiple sources, such as multiple regions (14).

In example (C), evidence combination may be primarily a question of system design. If the SyS aim is to monitor cases in humans, using the chicken cases as a predictor will actually explain a lot of the variability in the number of observations, reducing the chances of an alarm. It is a good explanatory statistical model, but a poor fit for SyS goals. A better option might be to develop a predictive model that uses the chicken data to foresee when human cases are likely to start increasing. This will however depend on having access to both of these data sources continuously and in a timely manner. When data sharing is not possible, alternatives can be sought by considering this explicitly as a DSS problem. What is the main decision we are trying to support? If this is preparedness to act in the case of a human outbreak, it may be enough to monitor the chicken time-series independently. Results from this monitoring process would then be continuously transmitted to public health officials.

Consider now example (B). As noted earlier, the farm-level indicators will be a combination of discrete and continuous time-series. Statistically, this poses a challenge to parallel monitoring. As the number of potential indicators at the farm level is high, we must find a way to combine their evidence; otherwise, users are left with a myriad of daily/weekly alarms that they will find difficult to interpret. To add complexity, statistical analyses must take into account predictors at different levels. In a single farm, monitoring an indicator of reproductive performance, for instance, may demand consideration of the age/parity of sows. This is not trivial, as typical syndromic indicators are grouped by unit of time, and therefore TADA can typically only handle variables that can be summarized per time point.

Making sense of multiple sources of evidence, all of which contribute to situational awareness around the same problem, remains an open area of research. If surveillance is framed as a problem of DSS design, the solution may not (only) be statistical. Rather, it involves a better understanding of the decisions we aim to support, and how each of the pieces of information generated can be used in that decision process. This will require intensive social research involving all stakeholders in the network; or, in DSS implementation terms, several rounds of iteration with users.

### 3.4. Interpreting alarms–the decision-making process

In order to start involving stakeholders in rounds of system implementation in practice we are missing one essential component of a DSS: the user interface. Discussions around dashboards for visualization of times-series analyses often stumble on a disconnection between the expertise of those who perform the analyses, and understand their outputs, and the experience of decision-makers.

The DSS approach suggests that the solution is to construct the simplest dashboard we can, and be prepared to iterate through the entire continuum, from data ingestion to output visualization, continuously, with direct user involvement. Decision-makers are not invited to design the system abstractly, but to use the system and give feedback based on one simple question: “how could this better support your decision-making process?” (15).

This approach assumes that implementation is context-based, which then leaves one main question–what is the decision-making process that we are primarily trying to support? “Early disease warning” may be too vague a goal to inform concrete design and implementation choices. As Fricker cautioned in his seminal paper in 2011 (2): “Looking for everything means it is harder to find any one thing.”

Phrasing decisions in a common language which both system designers and users are familiar with will likely require narrowing down to concrete threats. This may mean that we design systems not to “detect emerging diseases” but which can, for instance, “provide an early signal of the introduction of PRRS (Porcine Reproductive and Respiratory Syndrome) in this specific region.” While the focus on specific diseases seems to go against the general preparedness that SyS was intended to address, it enables us to move forward with the practical implementation of real-world applications, which support surveillance in practice. It will bring stakeholders together and establish collaborative practices that can be used to gradually expand system goals, and address an increasing number of decision scenarios.

## 4. Conclusion

The data-driven focus of SyS to date has resulted in times-series analyses being applied to the data at hand, without sufficient consideration being given as to the key questions such analyses should be attempting to answer. Implementation in practice will require that we define the following: who are the decision makers?; what specific problems they are trying to handle?; and how will information that supports their decisions can be delivered in consumable ways? The field of decision support systems design suggests that the main goal should not simply be, “getting the right information to the right person at the right time,” but that “the ultimate objective must be viewed in terms of the ability of information systems to support the improved performance of people in organizations” (7). We suggest that a DSS approach to SyS system design will help solve many of the current methodological challenges, in particular those associated with combining numerous and varied sources of evidence as well as assisting users to make sense of complex system outputs.

## Data availability statement

The original contributions presented in the study are included in the article/supplementary material, further inquiries can be directed to the corresponding author.

## Author contributions

FD wrote a first article abstract, which all authors reviewed and approved. All authors contributed to the thoughts developed in this article and actively engaged in discussions to mature the ideas proposed.
